# Awareness and uptake of herpes zoster vaccine among patients with diabetes mellitus in the Aseer Region, Saudi Arabia: A cross-sectional study

**DOI:** 10.1097/MD.0000000000044356

**Published:** 2025-09-05

**Authors:** Ali Alamri, Ahmad Althaqafi, Rabia Salawati, Raghad Asiri, Maha Aldugman, Majed Al Saleh, Bandar AlAsmari, Meteb AlBraik, Ramy Mohamed Ghazy

**Affiliations:** a Family Medicine, Aseer Central Hospital, Abha, Saudi Arabia; b Family and Community Medicine, College of Medicine, King Khalid University, Abha, Saudi Arabia.

**Keywords:** diabetes mellitus, health service utlization, herpes zoster, Saudi Arabia, vaccine hesitancy

## Abstract

Herpes zoster (HZ) is a reactivation of the varicella-zoster virus. It is a common infection, especially among patients with diabetes mellitus (DM). This study aimed to assess the awareness and uptake of the HZ vaccine, as well as their determinants, among patients with diabetes DM in the Aseer Region, Saudi Arabia. An anonymous cross-sectional study was conducted using a self-administered questionnaire between July 1, 2024 and October 31, 2024. We randomly included participants aged 18 years or older with DM. The questionnaire addressed socio-demographic characteristics, medical history regarding HZ, diabetes control, awareness, perception, and uptake practices regarding the HZ vaccine. A total of 200 participants were included: 34.5% were aged over 50 years, 51.0% were males, 90.5% were Saudi, 68.0% were married, 40.5% held a university degree, 67.0% were nonsmokers, and 57.0% had chronic diseases other than DM. Of the studied participants, 18.5% reported a previous HZ infection. Older age, smoking, and comorbid chronic illnesses were significantly associated with HZ infection. A majority expressed positive perception toward the vaccine’s effectiveness (77.0%), safety (82.5%), and side effects (80.0%). However, only 21.5% had actually received the vaccine. Predictors of awareness were being aged over 50 years (adjusted odds ratio [aOR] = 4.44, 95% confidence interval [CI]: 1.36–14.54, *P* = .014), divorced individuals [aOR = 12.70, 95% CI: 1.52–106.43, *P* = .019), and being nonsmokers (aOR = 0.36, 95% CI: 0.16–0.81, *P* = .014). The perceived side effects of the vaccine emerged as a significant predictor of vaccine uptake. Specifically, participants who believed the vaccine causes no side effects (aOR = 22.37, 95% CI: 2.31–216.32, *P* = .007). This study reveals a concerningly low vaccine uptake despite high levels of awareness and positive perception toward its effectiveness and safety. Efforts to maintain the high awareness and promote vaccination are needed.

## 1. Introduction

Varicella-zoster virus (VZV) or human herpes virus 3 (genus alpha herpesviridiae) is a double-stranded deoxyribonucleic acid virus belonging to the alpha herpes virus subfamily, is the causative agent of both varicella (chickenpox) and herpes zoster (HZ) or shingles.^[1]^ Varicella is transmitted through inhalation of respiratory droplets or direct contact with fluid from vesicular skin lesions. HZ is a reactivation of the VZV, which remains dormant in the sensory ganglia after a primary chickenpox infection.^[2]^ The average lifetime risk of HZ in developed countries is estimated to be about 30%.^[3]^ VZV reactivation results from declining cell-mediated immunity, making HZ a particular concern among older adults, individuals with immunocompromising conditions, or those receiving immunosuppressive treatments, as it may lead to more severe and complicated disease manifestations.^[4]^ HZ reactivation has been reported as a potential adverse event following coronavirus disease 2019 vaccination as well.^[1]^

HZ is characterized by the presence of painful, unilateral vesicular rash that can lead to severe complications.^[5]^ In addition, herpes zoster ophthalmicus, which involves the ophthalmic branch of the trigeminal nerve, occurs in approximately 10% to 20% of cases and may lead to ocular symptoms and potential vision loss.^[1]^ Although the rash and pain associated with HZ usually resolve within a month, pain can be severe and persistent, sometimes resulting in a complication known as postherpetic neuralgia (PHN), where pain continues long after the rash has healed.^[6]^ PHN can last for months or even years, causing chronic pain that interferes with daily activities, sleep, and overall well-being.^[7]^ Additionally, HZ has been associated with vasculitis (contributing to significant morbidity and mortality) and may also elevate the risk of cardiovascular events.^[1,8]^ Besides physical complications, studies have consistently demonstrated that individuals with HZ are at an increased risk for psychological distress, including anxiety and depression.^[9]^ A study by Chen et al,^[10]^ found that those with HZ had a higher incidence of developing major depression (2.2% vs 1.4%) and depressive disorders (4.3% vs 3.2%) than did the control group. Furthermore, Schmader et al,^[11]^ reported that the quality of life (QoL) in patients with HZ is significantly reduced, with notable declines in mental health-related QoL measures. The psychological burden of managing chronic pain, coupled with the fear of recurrent episodes, contributes to the overall mental health challenges faced by these patients.^[12]^

The impact of these symptoms highlights the critical need for preventive measures, such as vaccination, to reduce the burden of HZ, particularly high-risk groups like patients with diabetes melitus (DM).^[13]^ HZ vaccines have been proven to be highly effective in preventing HZ and its complications.^[14]^ Currently, 2 vaccines are available for HZ. The live attenuated zoster vaccine has been shown to be safe and moderately effective, reducing the incidence of HZ by 51.3% and PHN by 66.5%. However, its efficacy declines with increasing age (particularly in individuals over 70) and diminishes over time. In contrast, the recombinant zoster vaccine has demonstrated superior efficacy, reducing HZ incidence by 97.2% in adults over 50 and by 91.3% in those over 70. It also reduced PHN by 88.8%, with no observed decline in effectiveness among individuals over 80 or during a 10-year follow-up period.^[15]^ Vaccination is recommended for adults aged 50 and older, as well as those at increased risk, such as individuals with DM.^[16]^

DM, a chronic metabolic disorder, has attained epidemic levels worldwide.^[17]^ It is marked by insulin resistance and hyperglycemia and is linked to various complications, including infections, due to the compromised immune systems of affected individuals.^[18–20]^ According to the International Diabetes Federation, approximately 10.5% of adults aged 20 to 79 years were living with diabetes in 2021, with this number projected to rise to 46% by 2045.^[21]^ In Saudi Arabia, the prevalence of type 2 diabetes mellitus (T2DM) is particularly concerning, with rates among the highest in the world.^[19]^ Rapid economic development, urbanization, and lifestyle changes have contributed to a significant increase in diabetes cases over the past few decades.^[22]^ A 2021 report by the International Diabetes Federation indicated that the prevalence of diabetes in Saudi Arabia is approximately 17.7%, with around 4.2 million adults affected.^[23]^ Research has indicated that individuals with DM are more susceptible to HZ compared to the general population.^[24]^ A study by Hata et al.,^[25]^ reported that patients with DM have a 2.01 to 2.85 times higher risk of developing HZ. The chronic inflammation and vascular complications associated with DM further exacerbate the risk of HZ, highlighting the importance of vaccination to reduce the complications of HZ.^[26]^ Despite its proven efficacy, the HZ vaccine’s coverage rate remains suboptimal in various regions.^[27]^ Understanding the factors influencing vaccine uptake is essential for improving vaccination strategies and ensuring that high-risk populations, such as patients with DM, are adequately protected. This study aimed to assess the awareness and uptake of the HZ vaccine, along with the associated determinants, among patients with DM in the Aseer Region, Saudi Arabia.

## 2. Methods

### 2.1. Study design

An anonymous, cross-sectional, descriptive study was conducted from July 1, 2024 to October 31, 2024, at Family Medicine Clinics at Aseer Central Hospital, Abha, Saudi Arabia. The Aseer Central Hospital served 366,516 patients last year, including 17,232 outpatient visits, 119,088 emergency cases, 39,405 dental center patients, and 35,702 rehabilitation cases. Additionally, the hospital performed 8164 surgeries, 274,551 laboratory tests, and 96,721 radiology examinations.

### 2.2. Sample size and study participants

The sample size was calculated using the Epi-Info software statistical package (version 7.2.5, Centers for Disease Control and Prevention, Atlanta). The criteria used for sample size calculation were as follows: 95% confidence limit, 80% power of the study, expected prevalence of HZ vaccine coverage among patients with DM of 13.3%, and non-response rate of 10% (based on the pilot study findings). The minimum sample size based on the previously mentioned criteria was 197 participants. We rounded the sample to be 200. The Aseer Region diverse population, encompassing various socio-economic backgrounds and lifestyles, provides a comprehensive sample for the current study. The inclusion criteria for this study were adult patients with aged 18 years or older with a confirmed diagnosis of DM who attended Family Medicine Clinics at Aseer Central Hospital in Abha, Saudi Arabia. Participants were required to provide informed consent and demonstrate a willingness to participate. The exclusion criteria included patients with incomplete or missing medical records, individuals with contraindications to receiving the HZ vaccine, such as severe allergies to its components, and those unable or unwilling to complete the self-administered questionnaire. Additionally, individuals who received the HZ vaccine as part of clinical trials or outside the study period were excluded from the study.

### 2.3. Sampling technique

Participants were selected using a simple random sampling method. A comprehensive list of patients with attending family medicine clinics was compiled, and a random sample was selected from those presenting for follow-up visits using a computer-generated algorithm. Data collection was conducted through a self-administered questionnaire administered in a face-to-face format to ensure accurate and reliable responses.

### 2.4. Study outcomes

The primary outcomes of this study included the level of awareness of HZ vaccine, perception toward its safety, effectiveness, and side effects, and the actual uptake of the vaccine among patients with DM. Secondary outcomes involved identifying key socio-demographic (i.e., age, gender, marital status, and smoking) and behavioral determinants associated with awareness and vaccine uptake.

### 2.5. Pilot study

Prior to data collection, a pilot study was conducted with 30 participants to assess the clarity, feasibility, and applicability of the study tools, the time required to complete the questionnaire, response rate, and vaccination uptake. Based on the pilot study findings, necessary modifications and improvements were made to enhance the study tools before commencing data collection. The time required to fill the questionnaire was 12 to 16 minutes. Regarding vaccination, 4 patients received the vaccine with a response rate of 90%. Data of the piloted participants were not included in the analysis.

#### 2.5.1. Data collection tool

A self-administered questionnaire was developed to assess the awareness about the shingles vaccine and its uptake in Aseer region, Saudi Arabia. A panel of 4 physicians: comprising 2 family medicine specialists, 1 diabetologist, and 1 public health consultant, conducted a literature review to identify relevant items for the questionnaire. Each proposed question was independently rated by the reviewers on a 4-point scale (1 = not relevant, 4 = highly relevant). Only items receiving a rating of 3 or 4 by the majority of reviewers were included in the final questionnaire. The participants were given a clear and concise explanation of the study’s objectives. The questionnaire consists of 4 parts:

- The first part includes socio-demographic data (age, sex, marital status, nationality, educational level, smoking status, and history of chronic diseases).
- The second part includes medical history regarding HZ (previous VZV infection and its frequency, and previous suffering from HZ complications).
- The third part includes data regarding diabetes control (type of DM, duration of DM, type of medication, recent HbA1c level, and presence of DM-related complications).
- The fourth part of the questionnaire assessed participants’ awareness (e.g., whether they had heard about the HZ vaccine), knowledge about the appropriate age for vaccination (<50 years or ≥50 years), and their perception toward vaccination (e.g., perception regarding the vaccine’s safety for patients with diabetes, its ability to prevent complications of HZ, and vaccine side effects (whether permanent, temporary, or absent)). It also evaluated participants’ practice regarding the shingles vaccine (i.e., whether they had received it) and identified the source of the vaccination recommendation (physician, friends and relatives, or others (including social media)).

### 2.6. Ethical considerations

Ethical approval was obtained from the Research Ethics Committee of Aseer Health Affairs (National Registration Number with NCBE-KACST, KSA: H-06-B-091; Approval No: REC-8-5-2024).The study was executed according to the ethical standards in the 1964 Declaration of Helsinki and its later modifications. Informed consent was obtained from all the subjects involved in the study. They were assured of their strict confidentiality and anonymity.

### 2.7. Data management and analysis plan

The data were analyzed using the Statistical Package for the Social Sciences (SPSS 22.0 software, IBM Corp., Armonk) and Stata Version 14 (StataCorp LLC, College Station). Qualitative variables were prescribed using numbers and percent; the Chi-square test was used for analysis, or Fisher exact test & Monte Carlo exact test (if more than 20% of the expected cell value is <5). When a statistically significant overall Chi-square test was detected, pairwise comparisons of column proportions were conducted using the Bonferroni correction to control for type I error due to multiple comparisons. A multivariable logistic regression model was used to predict a dependent variable (awareness and vaccine uptake) based on socio-demographic variables, which had associations in bivariate analysis with a *P*-value <.2. Predictors were described using effect estimates with corresponding 95% confidence intervals (CIs). *P*-value (<.05) was adopted as the level of significance.

## 3. Results

Table 1 displays the socio-demographic and personal characteristics of the 200 study participants. Of them, 34.5% were over 50 years old, 51.0% were males, 90.5% were Saudi, 68.0% were married, 40.5% had a university education, 67.0% were nonsmokers, and 57.0% had chronic diseases other than DM. The majority of participants had T2DM (63.0%), followed by type I DM (29.5%) and gestational DM (7.5%). The duration of the disease varied, with 46.5% having DM for more than 5 years, 27.0% for 1 to 5 years, and 26.5% for 1 year or less. Regarding medication, 42.0% of participants were on oral medication, 25.5% on insulin injections, 18.0% used both, and 14.5% managed their diabetes with diet regimens alone. Glycosylated hemoglobin (HbA1c) levels were abnormal in 55.5% of participantsnormal in 30.5%, and were unreported or missing in 14.0%. Nearly three-fourths of the participants (76.0%) did not have diabetes-related complications.

**Table 1:** Socio-demographic and personal characteristics of the study participants.

Studied variables (N = 200)		N	%
Age (yr)	18–30	44	22.0
	31–40	31	15.5
	41–50	56	28.0
	>50	69	34.5
Sex	Female	98	49.0
	Male	102	51.0
Nationality	Saudia	181	90.5
	Non-Saudia	19	9.5
Marital status	Married	136	68.0
	Widowed	17	8.5
	Divorced	13	6.5
	Single	34	17.0
Completed educational level	Not educated	11	5.5
	Primary education	32	16.0
	Secondary education	58	29.0
	University education	81	40.5
	Postgraduate studies	18	9.0
Smoking status	Current smoker	37	18.5
	Previous smoker	29	14.5
	Nonsmoker	134	67.0
DM type	Type I	59	29.5
	Type II	126	63.0
	Gestational DM	15	7.5
Disease duration (years)	<1	53	26.5
	1–5	54	27.0
	>5	93	46.5
Type of medication	Oral medication	84	42.0
	Insulin injections	51	25.5
	Both	36	18.0
	Diet regimens	29	14.5
Recent glycosylated hemoglobin level	Controlled diabetes (<7%)	61	30.5
	Uncontrolled diabetes (≥7%)	111	55.5
	Unreported/missing	28	14.0
Presence of DM-related complications	Yes	48	24.0
	No	152	76.0
Having chronic diseases other than diabetes including obesity (yes)*		114	57.0
	Bronchial asthma	19	9.5
	Hypertension	50	25.0
	Immunological disorders	11	5.5
	Obesity	32	16.0
	Others	15	7.5

* Multiple response question.

**Figure 1.**
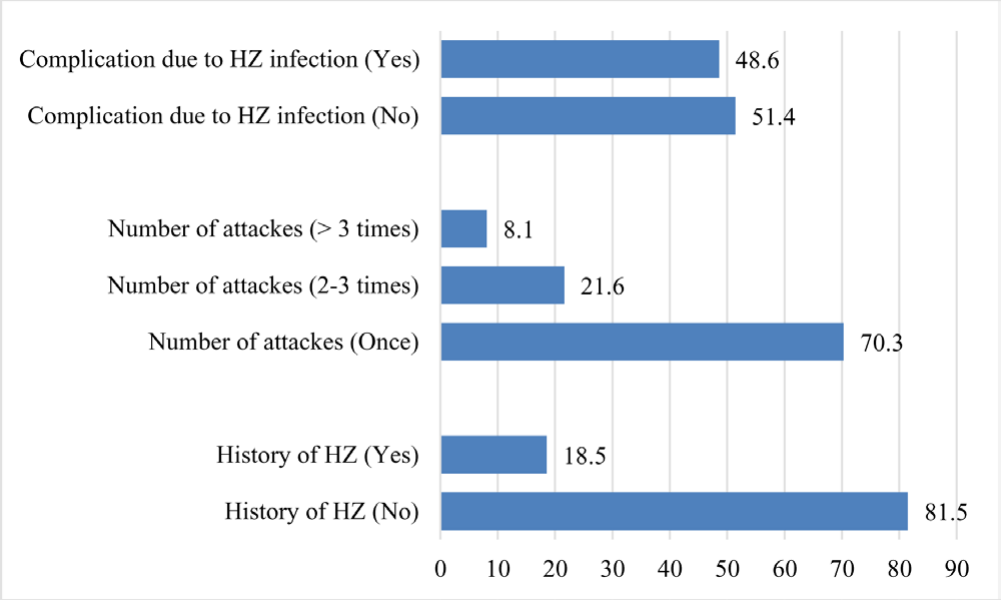
Medical history regarding herpes zoster infection among the study participants.

In total, 18.5% reported a previous history of HZ. Of those who had a prior infection, most (70.3%) had been infected only once, 21.6% had been infected 2 to 3 times, and 8.1% had experienced more than 3 infections. Regarding complications from HZ, 48.6% of those with a previous infection reported suffering from complications (Fig. 1).

Three variables showed statistically significant associations with HZ infection. First, age was significantly associated (*P* = .039), with post hoc analysis revealing that participants aged over 50 years had a markedly higher proportion of HZ infection (29.0%) compared to other age groups. Second, smoking status was significantly associated with HZ infection (*P* = .001). Previous smokers exhibited a substantially higher proportion of HZ infection (41.4%) than non-smokers (11.9%), while current smokers (24.3%) did not differ statistically from either group. Lastly, the presence of chronic diseases was also significantly associated with HZ infection (*P* = .004), as participants with chronic conditions had a higher proportion of HZ infection (25.4%) compared to those without such diseases (9.3%) (Table 2).

**Table 2:** Association between socio-demographic and personal characteristics and history of herpes zoster infection among the study participants.

Studied variable	Level	Yes (N = 37)	No (N = 163)	*P*-value
Age (yr)	18–30	4 (9.1%)ᵃ	40 (90.9%)ᵃ	.039*
	31–40	5 (16.1%)ᵃ	26 (83.9%)ᵃ	
	41–50	8 (14.3%)ᵃ	48 (85.7%)ᵃ	
	>50	20 (29.0%)ᵃ	49 (71.0%)ᵇ	
Sex	Female	17 (17.3%)	81 (82.7%)	.681
	Male	20 (19.6%)	82 (80.4%)	
Nationality	Saudi	30 (16.6%)	151 (83.4%)	.055^fe^
	Non-Saudi	7 (36.8%)	12 (63.2%)	
Marital status	Married	26 (19.1%)	110 (80.9%)	.091
	Widowed	5 (29.4%)	12 (70.6%)	
	Divorced	4 (30.8%)	9 (69.2%)	
	Single	2 (5.9%)	32 (94.1%)	
Education level	Not educated	2 (18.2%)	9 (81.8%)	.101
	Primary education	7 (21.9%)	25 (78.1%)	
	Secondary education	16 (27.6%)	42 (72.4%)	
	University education	8 (9.9%)	73 (90.1%)	
	Postgraduate studies	4 (22.2%)	14 (77.8%)	
Smoking status	Current smoker	9 (24.3%)ᵃ	28 (75.7%)ᵃ	.001*
	Previous smoker	12 (41.4%)ᵃ	17 (58.6%)ᵇ	
	Nonsmoker	16 (11.9%)ᵃ	118 (88.1%)ᵇ	
Chronic diseases	Yes	29 (25.4%)	85 (74.6%)	.004*
	No	8 (9.3%)	78 (90.7%)	

Superscript letters (ᵃ^,^ ᵇ) indicate that column proportions labeled with the same letter are not significantly different from each other at the .05 level (Bonferroni adjustment for multiple comparisons).

* Significant *P* < .05.

**Figure 2.**
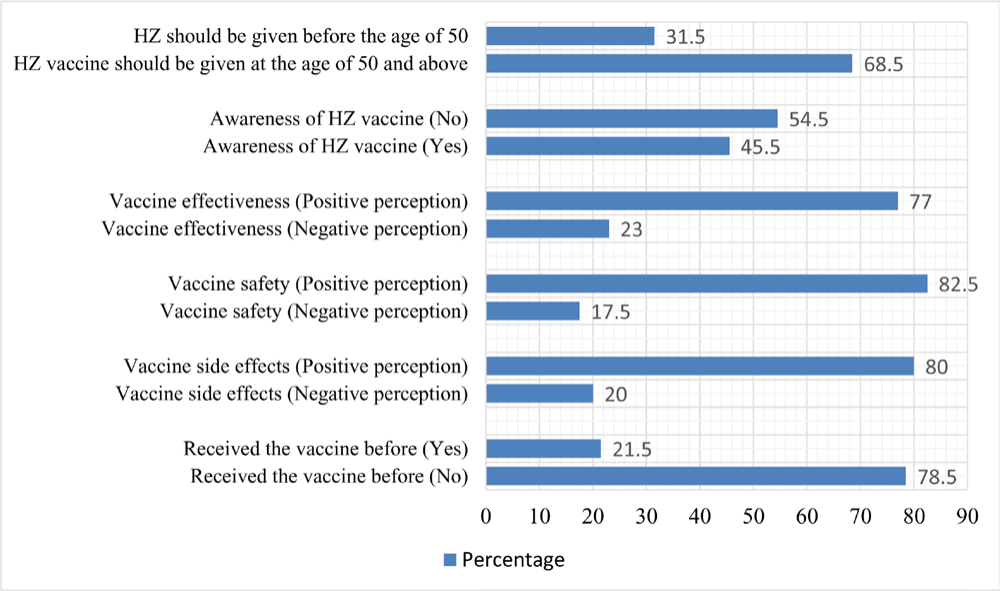
Awareness, behavior, and practices regarding the shingles vaccine among the study participants.

Figure 2 shows the study participants’ awareness, perception, and practices regarding the shingles vaccine. More than half of the participants (54.5%) were aware of the shingles vaccine and 68.5% knew that it should be given after the age of 50 years and above. A significant proportion of the participants held a positive perception towards the effectiveness of the shingles vaccine, with 77.0% expressing confidence in its efficacy. Similarly, 82.5% of the participants had a positive perception towards the safety of the shingles vaccine. The perception of the vaccine’s side effects was also largely positive, with 80.0% of the participants believing the side effects were minimal or manageable. Only 21.5% of the participants had received the shingles vaccine before.

Table 3 illustrates the association of socio-demographic and personal characteristics with awareness and uptake of the shingles vaccine among the study participants. Age was significantly associated with both awareness (*P* = .015) and receipt of the vaccine (*P* = .001). Older participants (>50 years) showed the highest awareness (69.6%) and highest vaccine uptake (33.3%). Post hoc analysis (Bonferroni) revealed that this group differed significantly in awareness from the youngest group (18–30 years), who had the lowest vaccine uptake (9.1%) and lower awareness (40.9%). Marital status showed a significant association with awareness (*P* = .013). Divorced participants had the highest awareness (92.3%), significantly different from single participants (47.1%) and widowed (70.6%) as indicated by different superscript letters. Smoking status was significantly associated with both awareness (*P* = .007) and practice (*P* = .003). Ex-smokers had the highest awareness (75.9%) and highest vaccine uptake (44.8%). Presence of chronic diseases was significantly associated with receipt of the vaccine (*P* = .024), with individuals having chronic conditions more likely to be vaccinated (27.2%) than those without (14.0%).

**Table 3:** Association of socio-demographic and personal characteristics with awareness and uptake of the shingles vaccine among the study participants.

Studied variables	Awareness of HZ vaccine		Received HZ vaccine	
	Aware (N = 109)	Not aware (N = 91)	Yes (N = 43)	No (N = 157)
Age (yr)				
18–30	18 (40.9%) ᵃ	26 (59.1%) ᵃ	4 (9.1%) ᵃ	40 (90.9%) ᵇ
31–40	15 (48.4%) ᵃ	16 (51.6%) ᵃ	1 (3.2%) ᵃ	30 (96.8%) ᵇ
41–50	28 (50.0%) ᵃ	28 (50.0%) ᵃ	15 (26.8%) ᵃ	41 (73.2%) ᵃ
>50	48 (69.6%) ᵃ	21 (30.4%) ᵇ	23 (33.3%) ᵃ	46 (66.7%) ᵇ
*P*-value	.015*		.001*	
Sex				
Female	55 (56.1%)	43 (43.9%)	17 (17.3%)	81 (82.7%)
Male	54 (52.9%)	48 (47.1%)	26 (25.5%)	76 (74.5%)
*P*-value	.652		.161	
Nationality				
Saudi	100 (55.2%)	81 (44.8%)	38 (20.9%)	143 (79.1%)
Non-Saudi	9 (47.4%)	10 (52.6%)	5 (26.3%)	14 (73.7%)
*P*-value	.512		.565^FE^	
Marital status				
Married	69 (50.7%) ᵃ	67 (49.3%) ᵃ	32 (23.5%)	104 (76.5%)
Widowed	12 (70.6%) ᵃ	5 (29.4%) ᵃ	5 (29.4%)	12 (70.6%)
Divorced	12 (92.3%) ᵃ	1 (7.7%) ᵇ	4 (30.8%)	9 (69.2%)
Single	16 (47.1%) ᵃ	18 (52.9%) ᵃ	2 (5.9%)	32 (94.1%)
*P*-value	.013*		.088^MC^	
Completed educational level				
Primary education	7 (63.6%)	4 (36.4%)	2 (18.2%)	9 (81.8%)
Secondary education	18 (56.3%)	14 (43.8%)	9 (28.1%)	23 (71.9%)
University education	29 (50.0%)	29 (50.0%)	15 (34.9%)	43 (65.1%)
Postgraduate studies	44 (54.3%)	37 (45.7%)	14 (17.3%)	67 (82.7%)
Not educated	11 (61.1%)	7 (38.9%)	3 (16.7%)	15 (83.3%)
*P*-value	.878		.622^MC^	
Smoking status				
Current smoker	24 (64.9%) ᵃ	13 (35.1%) ᵃ	8 (21.6%)ᵃ	29 (78.4%)ᵃ
Ex-smoker	22 (75.9%) ᵃ	7 (24.1%) ᵇ	13 (44.8%)ᵃ	16 (55.2%)ᵇ
Nonsmoker	63 (47.0%) ᵃ	71 (53.0%) ᵇ	22 (16.4%)ᵃ	112 (83.6%)ᵇ
*P*-value	.007*		.003*	
Presence of chronic diseases				
Yes	68 (59.6%)	46 (40.4%)	31 (27.2%)	83 (72.8%)
No	41 (47.0%)	45 (52.9%)	12 (14.0%)	74 (86.0%)
*P*-value	.092		.024*	

Superscript letters (ᵃ^,^ ᵇ) indicate that column proportions labeled with the same letter are not significantly different from each other at the .05 level (Bonferroni adjustment for multiple comparisons).

FE = Fischer exact test; MC = Monte Carlo test.

* Significant *P* < .05.

**Figure 3.**
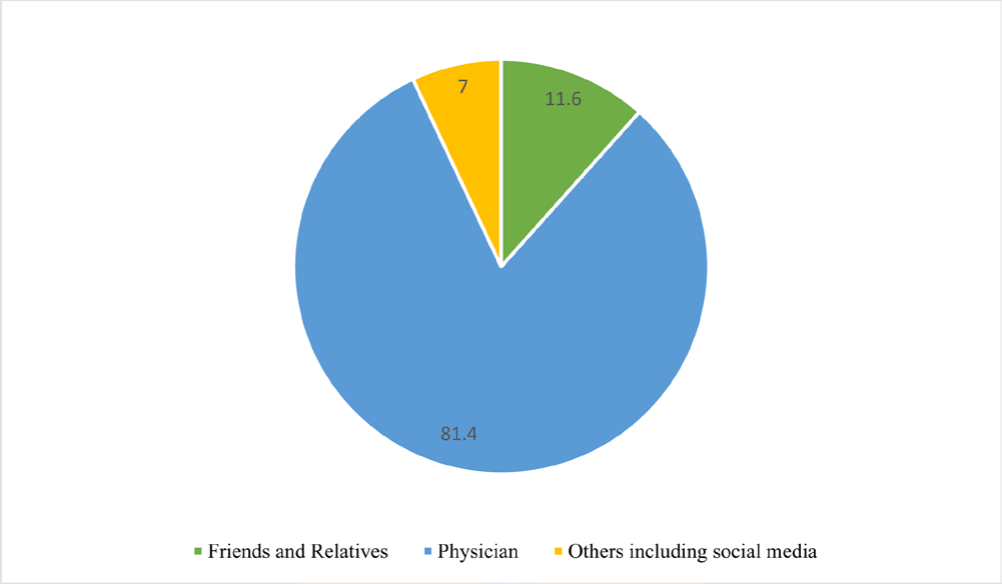
Sources of advice on receiving the herpes zoster vaccine.

The primary sources of advice regarding receiving the HZ vaccine were physicians (81.4%), followed by friends and relatives (11.6%), with “Others,” including social media, accounting for 7% (Fig. 3).

Table 4: participants who perceived the vaccine is safe were significantly more likely to have received it (24.8%) compared to those who did not (5.7%, *P* = .012). Similarly, perceptions of vaccine side effects showed a strong association with uptake (*P* < .001). Those who believed side effects are only temporary had the highest uptake (29.9%), whereas those who perceived side effects as permanent had the lowest (2.5%). Although perception in the vaccine’s ability to prevent complications showed a trend toward higher uptake (24.0% vs 13.0%), the association was not statistically significant (*P* = .112).

**Table 4:** Association between perception toward the vaccine and uptake.

Perception	Categories	Received the vaccine		*P*-value
		No N (%)	Yes N (%)	
Vaccine is safe	Agree	124 (75.2)	41 (24.8)	.012
	Do not agree	33 (94.3)	2 (5.7)	
Vaccine can protect from complications	Agree	40 (87.0)	6 (13.0)	.112
	Do not agree	117 (76.0)	37 (24.0)	
Side effects of the vaccine	Permeant	39 (97.5)a	1 (2.5)^b^	<.001^MC^
	There are no side effects	36 (83.7)a	7 (16.3)^a^	
	Temporal	82 (70.1)a	35 (29.9)^b^	

Superscript letters (ᵃ^,^ ᵇ) indicate that column proportions labeled with the same letter are not significantly different from each other at the .05 level (Bonferroni adjustment for multiple comparisons).

MC = Monte Carlo test.

* Significant *P* < .05.

Table 5 displays the logistic regression model results identifying possible predictors of awareness and vaccine uptake regarding the shingles vaccine among the study participants. In the first logistic regression model assessing awareness of the HZ vaccine among patients with diabetes, 3 variables were found to be statistically significant predictors. Participants aged over 50 years were significantly more likely to be aware of the vaccine compared to those aged 18 to 30 years (adjusted odds ratio [aOR] = 4.44, 95% CI: 1.36–14.54, *P* = .014). Additionally, divorced individuals had significantly higher adjusted odds of awareness compared to married participants (aOR = 12.70, 95% CI: 1.52–106.43, *P* = .019). Smoking status also showed a significant association, with nonsmokers being less likely to be aware of the HZ vaccine compared to current smokers (aOR = 0.36, 95% CI: 0.16–0.81, *P* = .014). In the second model examining predictors of HZ vaccine uptake among the same population, only the perceived side effects of the vaccine emerged as a significant predictor. Specifically, participants who believed the vaccine causes no side effects were significantly more likely to have received the vaccine compared to those who believed it causes permanent side effects (aOR = 22.37, 95% CI: 2.31–216.32, *P* = .007). Other variables such as age group, marital status, smoking status, chronic disease presence, and perceptions about vaccine safety and effectiveness did not show a statistically significant association with vaccine uptake after adjustment.

**Table 5:** Possible predictors using a logistic regression model for awareness & uptake regarding the shingles vaccine among the study participants.

Model 1: predictor of HZ vaccine awareness among patients with diabetes mellitus														
Predictor	B	SE		Wald		df			*P*-value			aOR		95% CI for OR
Age group				8.97		3			.03*					
31–40 vs 18–30	0.49	0.589		0.70		1			.404			1.64		0.52–5.19
41–50 vs 18–30	0.41	0.58		0.50		1			.48			1.51		0.48–4.70
>50 vs 18–30	1.49	0.605		6.07		1			.014*			4.44		1.36–14.54
Marital status				6.69		3			.083					
Single vs married	0.65	0.565		1.34		1			.247			1.92		0.64–5.81
Divorced vs married	2.54	1.085		5.49		1			.019*			12.70		1.52–106.43
Widow vs married	0.11	0.65		0.03		1			.87			1.11		0.31–3.98
Smoking status				9.87		2			.007*					
Nonsmoker vs current smoker	-1.03	0.419		6.01		1			.014*			0.36		0.16–0.81
Previous smoker vs current smoker	0.17	0.597		0.08		1			.775			1.19		0.367–3.82
Chronic diseases (yes vs no)	-0.12	0.338		0.13		1			.723			0.89		0.46–1.72
Constant	-0.04	0.615		0.01		1			.943			0.96		

Model 2: Predictor of HZ vaccine uptake among patients with diabetes mellitus														
Predictor			B		SE		Wald	df		*P*-value	OR		95% CI for OR	
Age group							6.798	3		.079				
31–40 vs 18–30			-1.36		1.27		1.17	1		.28	0.25		0.02–3.07	
41–50 vs 18–30			0.80		0.81		0.97	1		.325	2.22		0.45–10.87	
>50 vs 18–30			1.29		0.81		2.56	1		.11	3.63		0.754–17.57	
Marital status							1.07	3		.785				
Single vs married			-0.78		0.99		0.61	1		.435	0.46		0.07–3.22	
Divorced vs married			-0.07		0.75		0.01	1		.93	0.94		0.21–4.10	
Widow vs married			0.58		0.88		0.44	1		.507	1.79		0.32–10.06	
Smoking status							5.62	2		.06				
Nonsmoker vs current smoker			-0.55		0.57		0.94	1		.332	0.58		0.19–1.75	
Previous smoker vs current smoker			0.70		0.65		1.16	1		.281	2.02		0.56–7.28	
Chronic diseases (yes vs no)			0.56		0.46		1.46	1		.228	1.75		0.71–4.31	
Believes vaccine is safe (yes vs no)			-1.14		1.05		1.18	1		.277	0.32		0.04–2.51	
Believes vaccine prevents complications			-0.53		0.77		0.48	1		.488	0.59		0.13–2.64	
Side effects of HZ vaccine							8.69	2		.013*				
Temporal vs no side effects			2.27		1.22		3.47	1		.062	9.71		0.89–106.16	
Absent vs no side effects			3.11		1.16		7.20	1		.007*	22.37		2.31–216.32	
Constant			-4.15		1.56		7.08	1		.008	0.02			

For Model 1: Dependent variable: awareness about the herpes zoster vaccine (yes = 1, no = 0). Independent variables: age group, marital status, smoking status, and presence of chronic diseases. Model fit: omnibus χ² (9) = 30.70, *P* < .001; Hosmer–Lemeshow test χ² (8) = 1.55, *P* = .992; Nagelkerke *R*² = 0.19; overall classification accuracy = 67.7%, age group: 18 to 30, marital status: married, smoking status: current smoker, chronic diseases: no.

For Model 2: Dependent variable: uptake of herpes zoster vaccine (yes = 1, no = 0). Independent variables: age group, marital status, smoking status, presence of chronic diseases, belief in vaccine safety, belief in vaccine efficacy in preventing complications, and perceived side effects of the herpes zoster vaccine. Model fit: omnibus χ² (13) = 50.687, *P* < .001; Hosmer–Lemeshow test χ² (8) = 4.46, *P* = .814; Nagelkerke *R*² = 0.36; overall model classification accuracy not provided; –2 log likelihood = 149.89. Reference categories: age group: 18 to 30, marital status: married, smoking status: current smoker, chronic diseases: no, belief vaccine is safe: no, belief vaccine prevents complications: no, side effects of HZ vaccine: no side effects.

* Significant *P* < .05.

## 4. Discussion

DM is highly prevalent in Saudi Arabia and significantly affects patients’ well-being, self-efficacy, and QoL^[28]^ Due to its widespread impact on multiple body systems and its association with various acute and chronic complications.^[29,30]^ This study aimed to assess the awareness and uptake of the HZ vaccine, as well as their determinants, among patients with DM in the Aseer Region of Saudi Arabia.

### 4.1. Summary of the main study findings

The study found that 18.5% of participants had a history of HZ infection, primarily experiencing only 1 episode, with nearly half reporting complications. Factors associated with HZ infection included older age (over 50 years), smoking status, and having chronic diseases. Awareness of the HZ vaccine was present in slightly over half of the participants, with 68.5% knowing it should be given after age 50. Awareness predictors included being over 50 and divorced, while nonsmokers were less aware. Most participants had positive perception towards the vaccine, believing in its effectiveness and safety. Despite this awareness and favorable perception, vaccine uptake remained low, with the perception in the absence of side effects being the strongest predictor of vaccination. Physicians were identified as the main source of vaccination recommendations.

### 4.2. Interpretation of the study findings

#### 4.2.1. Infection with HZ

The current study found a relatively low incidence of HZ infection, reported at 18.5%.The high proportion of patients with elevated HbA1c levels (55.5%) suggests potential deficiencies in effective diabetes management, which may indirectly increase susceptibility to VZV reactivation. Supporting this, a retrospective study found that patients with poor glycemic control (reflected by a mean HbA1c of 10.7%) had a significantly higher risk of developing HZ.^[31]^ A meta-analysis also showed that patients with DM have a 1.6-fold increased risk of developing HZ. Additionally, it is estimated that about 13% of HZ cases occur in individuals with undiagnosed T2DM.^[32]^ This highlights the importance of routine diabetes screening among patients with HZ, as undiagnosed T2DM is often found present.^[33]^ Interestingly, a study conducted by Chen et al,^[34]^ suggested that type 1 DM may present a higher risk of HZ infection compared to T2DM. This high incidence of VZ among patients with DM may be due to the significant reduction in key immune functions, such as cell-mediated immunity, phagocytosis, and opsonization.^[35]^ Moreover, certain antidiabetic medications (such as thiazolidinediones, alpha-glucosidase inhibitors, dipeptidyl peptidase-4 [DPP-4] inhibitors, and insulin) have been associated with an increased risk of HZ, potentially due to their inhibitory effect on CD26, a key molecule involved in immune regulation.^[36]^ We also found a notable proportion of those infected experienced complications (48.6%). Similarly, PHN occurs more frequently in patients with DM, and zoster-related pain tends to be more severe in individuals with T2DM.^[12,37]^ These findings emphasize the need for proactive strategies in diabetic care, including increased clinical vigilance for HZ symptoms, and consideration of HZ vaccination, especially for high-risk diabetic subgroups. Vaccination recommendations should primarily come from physicians, as our findings indicate that most individuals who received the vaccine did so based on their physician’s advice.

### 4.3. Factors associated with HZ infection

In this study, the significant association between older age, smoking status, and the presence of chronic diseases with a history of HZ infection aligns with existing literature on risk factors for the disease. Regarding age, several studies found an increased HZ risk in 50 to 64-year-olds and ≥65-year-olds, highlighting them as dangerous, risky groups.^[38,39]^ In a Japanese cohort study, age ≥ 75 years, DM, dyslipidemia, hyperuricemia, hypertension, heart failure, and glucocorticoid administration were associated with increased risks of HZ onset.^[40]^ Moreover, hospitalization rates and mortality associated with HZ increase with age. Ongoing demographic shifts and population aging are expected to further elevate the global burden of the disease.^[37]^ This finding underscores the critical need for targeted preventive measures in older adults. Public health initiatives should prioritize HZ vaccination for high-risk groups, especially those over 50 years of age.

### 4.4. Awareness about vaccination

The current study revealed that while a significant portion (45.5%) of patients with DM were not aware of the vaccine. Older age was a significant predictor of vaccine awareness, which may be attributed to a heightened perception of risk among individuals in this age group. In the bivariate analysis, former smokers exhibited greater engagement in preventive health behaviors, as reflected by higher awareness and uptake of the HZ vaccine. This may be attributed to increased health consciousness, previous exposure to risk communication, or personal health concerns that motivated them to quit smoking and adopt proactive health practices. However, in the multivariable analysis, only nonsmoking status remained a significant predictor, and interestingly, it was associated with lower awareness. This unexpected finding may suggest residual confounding or differences in health messaging exposure between groups. This reported high awareness is consistent with the results of another study in Saudi Arabia, where 51.6% claimed to be aware of the HZ vaccine.^[41]^ On the other hand, these findings are slightly lower than in another study in Korea, where the participants revealed a high awareness of HZ (85.4%).^[42]^ This discrepancy suggests that cultural, healthcare infrastructure, and public health communication differences may influence vaccine knowledge. Addressing these gaps through targeted educational campaigns could improve vaccine uptake and reduce HZ-related complications among diabetic populations.

### 4.5. Vaccine uptake

The current study found that although over half of the participants were aware of the vaccine, only 21.5% had received it. The study’s finding of a gap between vaccine awareness and actual uptake emphasizes the importance of identifying potential barriers to vaccination, such as restricted access, questions about vaccine safety or efficacy, or a lack of strong healthcare practitioner recommendations. Our findings are consistent with the results of another Saudi study on patients with diabetes, where 1-quarter of the participants intended to receive the HZ vaccine.^[43]^ Also, the Greece study reported a coverage rate of 26.3% among individuals with diabetes aged 60 years and older.^[44]^ HZ vaccination coverage in different countries has been noted to vary. Coverage rates of 9%, 11.9%, and 16.57% have been reported in South Korea, Texas, and China, respectively.^[42,45,46]^ This gap between population awareness and practice was observed for other vaccines like the human papillomavirus vaccine.^[47]^ This disparity emphasizes the necessity for focused programs that not only raise awareness but also address the issues that hinder people from being vaccinated.

Our study found a high prevalence of positive perceptions regarding the vaccine’s effectiveness and safety. In bivariate analysis, we identified a significant association between patients’ perceptions and their decision to receive the vaccine, particularly concerning beliefs about vaccine safety and the absence of side effects. Notably, the belief that the vaccine causes no side effects emerged as the only significant predictor of vaccine uptake in multivariable analysis. Several studies have reported similar findings for other vaccines, including the coronavirus disease 2019 vaccine^[48]^ and monkeypox vaccines.^[49]^ These findings underscore the critical role of perception in shaping vaccination behavior. From a public health perspective, this highlights the need for targeted communication strategies that specifically address concerns about vaccine safety and clearly explain the nature and frequency of potential side effects.

### 4.6. Implications of this research

The findings of this study have important implications for public health policies aiming at increasing shingles vaccination awareness and uptake, particularly among high-risk groups such as patients with DM in Saudi Arabia. Participants above the age of 50, smokers, and those with chronic diseases were found to be at high risk for HZ infection among diabetes patients. Public health programs should emphasize these populations, offering appropriate information and resources to encourage immunization. The high prevalence of HZ problems among these groups emphasizes the importance of implementing targeted protection efforts. Healthcare professionals should incorporate vaccine information into diabetes treatment programs, stressing the elevated risk of HZ among patients with poor glycemic control. The findings of high knowledge despite low vaccine uptake imply the need for increasing the accessibility and convenience of vaccination. This could include establishing vaccination clinics in community centers as 1 of the community-based initiatives to control diabetes and its complications,^[50]^ providing vaccines during routine medical appointments, and ensuring that vaccines are provided for free or at a reduced cost.

## 5. Limitations and strengths

This study has several limitations that should be acknowledged. First, its cross-sectional design restricts the ability to establish causal relationships between independent variables (e.g., socio-demographic factors) and outcomes such as vaccine awareness and uptake. The reliance on self-administered questionnaires introduces potential reporting bias, as participants may underreport or overreport their symptoms and the frequency of HZ recurrence. For instance, this limitation is evident in the unexpectedly high proportion of participants reporting a diagnosis of type 1 DM. Additionally, while the demographic focus on the Aseer region provides valuable insights, the findings may not be entirely generalizable to other regions. However, the study also has notable strengths. By focusing on patients with diabetes (a group at higher risk for HZ) the research provides specific insights that can inform targeted public health interventions. Encompassing various detailed socio-demographic information, medical history, and vaccination practices of patients with diabetes.

## 6. Conclusions

In conclusion, our study highlights a concerning diagnosis of HZ among patients with diabetes in Saudi Arabia, with low uptake of the vaccine despite a higher magnitude of awareness. The findings reveal a significant gap between the level of awareness and actual vaccine uptake. By addressing the identified gaps and incorporating comprehensive vaccine care information in diabetes management, we can improve the overall QoL for patients with DM in Saudi Arabia. Older age, smoking, and marital status were significantly associated with awareness of the HZ vaccine, while believing that the vaccine has no side effects was a significant predictor of a positive perception toward vaccination.

## Acknowledgments

The research team expressed unlimited gratitude to the respondents who gave us their time and agreed to participate in our study. The research team expresses their deep thanks to the study participants for providing their time and sharing in this study. The authors extend their appreciation to the Deanship of Research and Graduate Studies at King Khalid University for funding this work through a Large Research Project under grant number RGP2/348/46.

## Author contributions

**Conceptualization:** Raghad Asiri, Metab AlBraik.

**Data curation:** Majed Al Saleh.

**Formal analysis:** Ramy Mohamed Ghazy.

**Investigation:** Ahmad Althaqafi.

**Software:** Maha Aldugman.

**Validation:** Rabia Salawati.

**Writing – original draft:** Bandar AlAsmari.

**Writing – review & editing:** Ali Alamri.
